# Federated MobileNetV2 with ensemble meta-learning for privacy-preserving brain tumor classification

**DOI:** 10.1038/s41598-026-55847-5

**Published:** 2026-06-04

**Authors:** Menali Paul, Ankitesh Tiwari, Bhavesh Barmashe, Kalpana Rai, Vikas Singh Panwar

**Affiliations:** 1 Department of Artificial Intelligence and Machine Learning, Sagar Institute of Research and Technology (SIRT), Bhopal, India; 2 Manipal Institute of Technology, Manipal Academy of Higher Education, Manipal, Karnataka India

**Keywords:** Federated learning, MobileNetV2, Ensemble meta-learning, FedAvg, FedProx, Brain tumor classification, Convergence analysis, Privacy-preserving deep learning, Medical imaging, Cancer, Computational biology and bioinformatics, Engineering, Mathematics and computing

## Abstract

The identification of brain tumors from MRI images is very crucial for the selection of an appropriate treatment. However, the existing solution has issues with privacy and data sharing. To address this challenge, this paper proposes the use of federated learning. The proposed solution employs a light convolutional backbone and some adaptive local meta-learners. The proposed solution employs MobileNetV2 as the feature extractor. This is fine-tuned for many clients using a combination of FedAvg and FedProx regularization. Each client also trains a few meta-learners (MLP, SVM, and ELM) using the local feature embeddings, enabling people to obtain personalized predictions without sharing their private information. For inference, the framework supports both probability-level averaging across client ensembles and deployable single-client prediction using only the local meta-learners of one client. On the Brain Tumor MRI Dataset, containing 7023 image slices across glioma, meningioma, no tumor, and pituitary classes, the proposed framework achieved a maximum observed accuracy of 99.57%. Across four repeated runs, it achieved 99.29% +/- 0.20% accuracy with a 95% confidence interval of 98.97% to 99.61%, while maintaining strong macro-F1 performance and a macro-average ROC-AUC of 0.998690. Under the same preprocessing and split protocol, it outperformed internally reimplemented CNN+FedAvg and CNN+FedAvg+FedProx baselines and preserved near-centralized ROC-AUC performance. Communication analysis showed that exchanging the MobileNetV2 backbone required 149.89 MB per round for five clients, corresponding to an 83.34% reduction relative to a ResNet-50 backbone.

## Introduction

The challenge with brain tumors is that they develop fast and can lead to severe damage to the brain if they are not identified on time. MRI is the primary non-invasive technique for identifying any issues within the skull, providing a detailed image of the brain, which is critical for identifying tumors. Recently, there have been breakthroughs in deep learning that have enabled the development of automated solutions capable of interpreting MRI scans with a high degree of accuracy, even better than human specialists^1,2^. Despite the progress made, the majority of the models for diagnosis are still centralized in terms of training, which involves pooling data from a variety of hospitals or imaging centers into a single database. This is difficult due to privacy and confidentiality issues^3,4^. MRI is widely used for brain tumor diagnosis due to its superior soft tissue contrast and non-invasive nature.

Federated Learning (FL) is all about model training on multiple distributed data sources without actually transferring the sensitive information. Multiple participants collectively update a common model by performing local updates on their respective data sources and then aggregating the model parameters on a central server^3,5^. Recent studies have highlighted the increasing role of federated learning in medical imaging for enabling collaborative analysis while preserving patient privacy^6,7^.

In practical scenarios, problems such as heterogeneous data types are frequently encountered, class imbalances, and non-convergence. Techniques such as FedAvg only aggregate updates for global updates, but they tend to perform poorly on client data with large variations^5^. Variants such as FedProx introduce a penalty term to reduce the effect of client drift and improve convergence for non-IID data^8^.

FL trains models with privacy in mind, but having one shared backbone may make it difficult for the model to learn the local characteristics of data from different institutions. To address this, this paper proposes a simple hybrid approach: a shared feature extractor and flexible local meta-learners. A MobileNetV2 backbone is shared and optimized for all clients to learn imaging features. Meanwhile, each client trains its own meta-learners, including an MLP, an SVM, and an ELM, based on the embeddings of their own data. This allows each client to maintain its own decision boundary while maintaining patient privacy.

In inference, the model relies on local strengths: the shared network identifies key features in the test data, and the meta-learner of each client provides probability scores. The scores are then combined to produce the final prediction of the ensemble. The hybrid method reduces communication requirements, is independent of data variations, and increases accuracy in classification in a distributed environment^9^.

The main contributions of this study consist of:

1. A decoupled federated-personalized framework in which MobileNetV2 learns a shared MRI feature representation, while client-side MLP, SVM, and ELM meta-learners provide local decision adaptation without communicating meta-learner parameters.
2. A transparent cross-silo simulation and evaluation design that separates global representation learning, local decision personalization, all-clients-online inference, and deployable single-client inference, enabling the framework to be evaluated under both collaborative and practical hospital-side inference settings.
3. An internally controlled empirical comparison under the same preprocessing, image-level split, and federated protocol, including centralized MobileNetV2, CNN+FedAvg, and CNN+FedAvg+FedProx baselines.
4. A comprehensive validation package including repeated-run evaluation, 95% confidence intervals, paired statistical testing, ROC-AUC analysis, communication-cost quantification, deployable single-client inference evaluation, and ablation analysis of MobileNetV2, FedProx, and each local meta-learner.

**Fig. 1:**
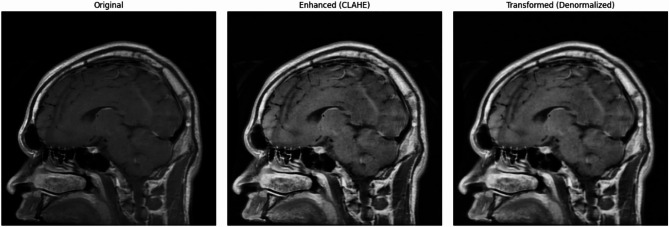
Pre-processing pipeline showing original MRI, CLAHE-enhanced MRI, and transformed image after resize and normalization.

**Fig. 2:**
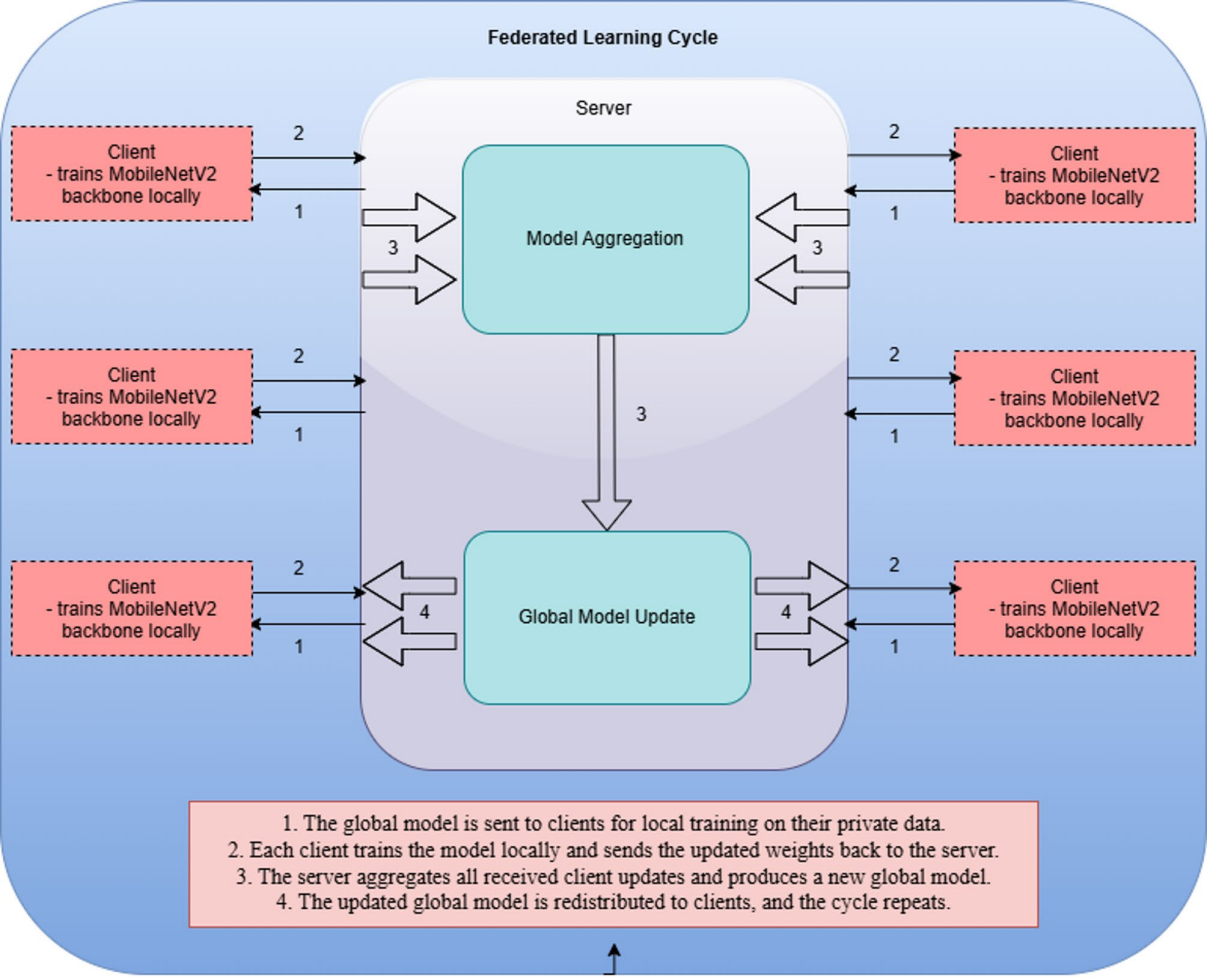
Federated learning process flow.

The novelty of the proposed framework lies in this decoupled federated-personalized design. Rather than relying only on a single global classifier or personalizing large neural-network components, the framework learns a shared MobileNetV2 MRI representation through federated optimization and performs client-side decision adaptation using lightweight MLP, SVM, and ELM meta-learners trained on local embeddings. This separates global representation learning from local decision modeling, keeps the meta-learner parameters private to each client, and avoids additional federated communication beyond the shared backbone updates. The single-client inference results and ablation analysis further support this design by showing that the framework remains deployable when only one client uses its local meta-learners, while MobileNetV2 provides the main representation gain and the meta-learner ensemble provides decision-level refinement (Figs. 1, 2).

### Overview and objectives

The proposed framework follows a federated learning workflow in which a shared MobileNetV2 backbone is distributed to multiple clients for decentralized training. Each client performs local optimization using the FedProx loss function to support local-update stability, while FedAvg is used to aggregate model updates at the server. At each federated round, the current global backbone is used to extract feature embeddings, and client-specific meta-learners are trained locally, allowing them to continuously adapt to the evolving feature space. During inference, predictions can either be combined across all clients through probability-level averaging or generated locally using a single client’s meta-learners with the shared global backbone.

The objective of this study is to develop a lightweight and privacy-preserving federated learning framework for classifying four types of brain tumors: glioma, meningioma, pituitary tumor, and no tumor from decentralized MRI data. Specifically, the study aims to evaluate the effectiveness of MobileNetV2 as a global feature extractor, assess the role of FedProx in stabilizing local updates during federated optimization, analyze the contribution of ensemble meta-learning in improving client-specific predictions, demonstrate high diagnostic performance using accuracy, F1-score, ROC-AUC, privacy, and communication-efficiency metrics, and evaluate convergence behavior across multiple federated rounds using macro-average performance metrics and confusion matrix analysis.

Recent federated learning approaches such as FDRL^10^ and FecMap^11^ focus on global representation learning and feature alignment across clients. The proposed framework differs by keeping the MobileNetV2 representation shared while using client-side meta-learners for local decision adaption without transmitting meta-learner parameters. This enables flexible and privacy-preserving personalization without additional FL payload from local meta learners, while improving stability under simulated federated participation. Unlike FDRL and FecMap, which primarily focus on improving global representation learning or feature alignment, the proposed framework introduces a decoupled architecture that separates global feature extraction from local decision modeling. The use of multiple client-side meta-learners enables diverse and adaptive local decision boundaries, which is not explicitly addressed in these methods.

In addition to preserving privacy, the proposed approach enhances the adaptability and effectiveness of local models by enabling client-specific meta-learners to capture unique data characteristics, resulting in improved performance under the evaluated federated simulation.

## Literature review

Brain tumor classification remains a very challenging task in analysing medical images due to the large variability of tumors in shape, appearance, and development. Recently, deep learning, particularly CNNs, has demonstrated potential in automatically classifying brain tumors from MRI images with high sensitivity and strong consistency. Xie et al.^2^ provided a general overview of the development of brain tumor classification using CNNs and the increasing need for interpretability in practical applications. The authors identify the prevailing trend: CNNs can be extremely accurate, but they are “black-box” models that are difficult to interpret.

In addition to CNN-based approaches, recent studies have explored transformer-based models, hybrid deep learning architectures, and traditional machine learning techniques for brain tumor classification. Vision transformers and hybrid CNN-transformer models have demonstrated strong performance in capturing global contextual features, while classical methods such as SVM and ensemble learning have been effectively combined with deep features to improve classification robustness. These diverse approaches highlight the evolving landscape of brain tumor analysis beyond conventional CNN-based models.

In addition, Capsule Networks, such as ConvCaps Capsule Networks and BayesCap, have been explored for brain tumor classification, offering equivariance to spatial transformations and improved modeling of hierarchical relationships between features. These models are particularly beneficial in medical imaging, where tumor structures may vary in orientation and shape^12^. Furthermore, uncertainty-aware variants such as BayesCap incorporate probabilistic representations, enabling improved reliability and confidence estimation in classification outcomes^13^.

Recent surveys on federated learning in medical imaging highlight its growing importance in enabling privacy-preserving collaborative model training across institutions. Similarly, comprehensive reviews on brain tumor classification using deep learning emphasize the need for robust and interpretable models for clinical deployment^14,6,7^. Comparative studies on federated learning methods for medical image analysis, such as COVID-19 detection, demonstrate the importance of robust optimization techniques under heterogeneous data distributions^15^. Recent approaches have addressed heterogeneity in medical federated learning through advanced representation alignment techniques, including vision transformer-based methods^16^.

Developments in CNN-based tumor classification are making deep learning (DL) models more robust and better at distinguishing different tumors. Mohan et al.^1^ proposed a new CNN configuration and compared it with VGG16, demonstrating that it performed better in distinguishing primary from metastatic tumors and that the idea of a deeper feature extractor being more useful in medical image analysis is valid. Similarly, Gumaei et al.^17^ proposed a hybrid approach that combines multi-layer feature extraction with regularized extreme learning machines, demonstrating that a combination of hand-crafted and deep features can significantly improve classification performance. Azzahra et al.^18^ evaluated the Inception-ResNet-V2 model for classifying several types of tumors and obtained excellent performance in distinguishing glioma, meningioma, and pituitary tumors. These studies, together with other CNN- and DNN-based MRI classification studies^19,20^, demonstrate how rapidly DL models for BTC are evolving on various MRI datasets.

Because of the sensitivity of medical information and the many regulations surrounding it, Federated Learning (FL) has emerged as a strong approach to train deep learning models without having to aggregate all the information in one place. Sheller et al. demonstrate that FL enables collaboration among multiple institutions without compromising patient privacy, providing strong evidence that training can be equally effective even when done in a decentralized manner. Further leveraging this, Yang demonstrated the applicability of FL to brain tumor analysis and demonstrated that privacy-preserving models can still achieve strong accuracy on MRIs from different institutions. The CyclicFL approach proposed by P. Zhang et al.^21^ improves federated optimization by taking advantage of cyclic model pretraining, which reduces the need for communication and stabilizes convergence. Wei Zhang et al.^22^ proposed an FL-based framework for brain tumor segmentation, demonstrating that FL is not only effective for classification tasks but also for pixel-level tasks, which are extremely important in surgical and radiological applications. Recent advancements have also focused on addressing heterogeneity in federated medical imaging through improved optimization and representation alignment techniques, further enhancing model robustness in non-IID environments Darzi et al.^15^.

At the heart of FL, FedAvg, proposed by McMahan et al.^5^, remains the most popular aggregation algorithm due to its simplicity and efficiency for a large number of clients. However, FedAvg become less stable when the data is non-IID, and the class distribution or image quality varies significantly across different institutions, which is common in real-world applications. To address this issue, Li et al.^8^ proposed FedProx, an extension of FedAvg, which includes a proximal term to ensure that the local models do not drift too far apart and aid in the global convergence of the training process. Sivakumar et al.^23^ leveraged this FedAvg-FedProx combination to develop a federated CNN for BTC and demonstrated improved robustness and accuracy for varying data splits. Their work demonstrates the effectiveness of FL, combined with contemporary regularization techniques, in significantly enhancing the readiness for deployment of multi-hospital tumor diagnostics.

Aside from FL theory, there are many works that prove the use of diverse datasets and unique architectures is beneficial for BTC. Works such as Das et al.^24^ and Ravinder et al.^25^ prove that CNN classifiers and graph convolutional models trained on diverse imaging examples have high sensitivity and good generalization to new types of cancer. Hybrid models and physics-inspired models such as G-ResNet^26^ and BayesCap^13^ also prove that architecture change can lead to improvement. In general, these works show that the performance of BTC relies on good feature extraction and model integration across institutions, hence the need for a lightweight MobileNetV2 architecture with a federated FedAvg-FedProx optimization for high accuracy and privacy preservation. Recent work also explores collaborative transfer learning for BTC with different data distributions^27^.

Recent studies have increasingly focused on robust, privacy-preserving, and personalized variants of federated learning for healthcare. RobustHealth addresses privacy-preserving heterogeneous mobile health diagnosis^28^, while CBRFL studies Byzantine-resilient aggregation for unreliable or malicious client participation^29^. Recent personalized federated learning methods for computer-aided diagnosis and medical image classification commonly personalize local model parameters, classifier heads, prototypes, or site-specific feature representations to improve adaptation under institutional heterogeneity^30,31^. In contrast, the present framework does not personalize large portions of the neural network. It keeps a shared lightweight MobileNetV2 representation and performs local decision adaptation through client-side MLP, SVM, and ELM meta-learners trained on embeddings. This positions the proposed method between conventional global-model FL and full personalized FL: it preserves a common feature extractor while allowing local decision boundaries to adapt without transmitting local meta-learner parameters or increasing the federated backbone payload. Recent work has also examined Beyond medical FL, robust graph-based representation learning with sparse structure learning has also been explored for handling structured data variability^32^, robust FL under adversarial wireless environments^33^ and adaptive task embeddings for context-shift reduction^34^, which further motivates the need to position the proposed framework within recent robust and adaptive distributed learning literature.

## Problem statement

Brain tumor classification using MRI images is a reality in the current state of neurodiagnostic equipment, assisting in early diagnosis and decision-making in the clinical setting^1,2^. The traditional deep learning approach involves a whole lot of data collection to develop robust models; however, in the medical field, bringing all patient information under one umbrella is not possible, as it stands contrary to medical ethics and data privacy regulations^3^. As a result, MRI images remain dispersed in hospitals, diagnostic laboratories, and research institutions, making it difficult to determine the extent to which any given model can access information. This is problematic, as CNN-based brain tumor classification models require a whole lot of information within each class to generalize well across different imaging modalities^2^.

MRI images represent brain tissues based on intensity variations, enabling the identification of tumor regions through differences in texture, shape, and contrast. Tumors often appear as irregular structures with distinct intensity patterns compared to surrounding healthy tissue. These characteristics make MRI-based analysis critical for accurate tumor classification and clinical decision-making.

MRI is preferred over other imaging modalities such as CT and functional MRI (fMRI) for brain tumor classification due to its superior soft tissue contrast and ability to clearly delineate tumor boundaries without exposing patients to ionizing radiation. While CT is useful for detecting bone structures and acute hemorrhage, it provides limited contrast for soft tissues. fMRI, on the other hand, focuses on brain activity rather than structural abnormalities, making it less suitable for tumor classification tasks. Therefore, MRI remains the most appropriate modality for accurate tumor detection and classification.

Federated learning (FL) aims to address these challenges by enabling model training without sharing local patient data^3^. Despite the advantages, FL introduces several challenges. Firstly, the existence of variability in client datasets, including differences in scanner parameters, demographic representation, image contrast, and tumor rate, leads to non-IID data settings, which can significantly hamper the performance of conventional FL optimization algorithms, such as FedAvg^5^. In non-IID settings, local updates from clients may differ in opposite directions, resulting in deteriorated global convergence, irregular training dynamics, and decreased diagnostic accuracy^5^. The proximal term in FedProx mitigates this problem to some extent^8^, but it may not work well when classification problems require a significant degree of representation distinction among different tumor classes.

Although real multi-institutional federated learning commonly involves severe non-IID variation, the present experimental setup uses a class-balanced near-IID client simulation to ensure stable participation and controlled comparison. FedProx is retained as a proximal regularization term for local-update stability, but the current setup should not be interpreted as a severe label-skew or hospital-level non-IID stress test.

Second, in the context of limited devices, models such as MobileNetV2 are very effective, but their representation capability is normally lower when trained on varied datasets only^35^. The reason is that the number of parameters is lower, making it more difficult to learn general features from the particular MRI segments, especially when each dataset presents slight variations in tumor morphology. Therefore, in lightweight federated learning, there is a need for techniques to improve the representation capability of the models without increasing their complexity^35^.

Thirdly, the stability of convergence evaluation becomes more challenging in the context of decentralized training. Contrary to centralized models, which tend to enhance with each step, federated models are characterized by client-specific noise, shifting data distributions, and non-uniform participation rates per round. Due to these aspects, instead of focusing on the stability of convergence, it is necessary to examine the convergence process per round through the use of validation accuracy and macro-averaged precision, recall, and F1-score.

With these challenges in mind, the primary research question addressed in this study is: How can a federated MobileNetV2-based framework provide strong diagnostic performance, stable convergence, privacy preservation, and reduced communication cost while supporting client-side decision adaptation?

This objective is addressed through the following steps:

- Utilize a MobileNetV2 global backbone network as it is efficient and aligns well with the distributed model.
- Implement a collaborative learning strategy where robust features of various clients are combined to enhance the global model’s representational capability.
- Implement a FedAvg-FedProx hybrid optimization strategy to minimize the drift of various clients and enhance convergence properties for varying distributions^8^.
- Perform a comprehensive convergence study over twenty iterations to demonstrate the convergence properties of the model and its ability to provide accurate diagnostic results^23^.

The problem statement mentioned above provides a foundation for explaining the approach mentioned in this section.

## Proposed methodology

The proposed framework employs federated learning along with efficient meta-learning to design a privacy-preserving and flexible workflow for brain tumor classification.

The framework consists of three key modules, as illustrated in Fig. 4: (i) federated global feature learning using MobileNetV2, (ii) client-specific meta-learner training for local personalization, and (iii) ensemble-based global inference.

- Train a shared MobileNetV2 backbone via federated optimization.
- Train meta-learners independently on each client using extracted feature embeddings^9^.
- Perform ensemble inference by aggregating predictions from all clients^9^.

### Dataset and preprocessing

This study used the public Brain Tumor MRI Dataset for all the experiments. The dataset is pre-labeled for glioma, meningioma, pituitary tumor, and no tumor classes. All MRI images undergo a standardized preprocessing pipeline to ensure consistency across clients. Images were loaded in grayscale, resized to 224 × 224, enhanced using CLAHE with clipLimit = 2.0 and tileGridSize = (8,8)^36^, and normalized using mean = 0.5 and standard deviation = 0.5. Horizontal flipping and 90-degree rotation augmentation were applied only to training images.

During training, data augmentation techniques including Horizontal Flip (*p* = 0.5) and RandomRotate90 (*p* = 0.3) are applied to improve generalization. For validation and test datasets, only resizing and normalization are applied without augmentation. This preprocessing pipeline ensures robustness and consistency for medical image analysis in a federated setting.

Each client is assigned a roughly equal number of training samples to ensure balanced participation; therefore, the resulting client partitions are class-balanced and represent a near-IID federated simulation at the label level. Minor feature-level variation may still arise from the randomly assigned MRI images, but the current setup should be interpreted as a class-balanced federated simulation rather than a severe non-IID label-skew setting.

The present client partition is class-balanced and near-IID at the label level. FedProx is retained because it is designed to improve local-update stability when client drift arises, particularly in more heterogeneous or severe non-IID federated settings. However, the current balanced partition should not be interpreted as a direct stress test of FedProx under severe non-IID hospital heterogeneity.

The experiments use the Brain Tumor MRI Dataset originally released by Masoud Nickparvar on Kaggle under the CC0: Public Domain license^37^. The GitHub repository listed in the Data Availability statement is used only as an access/redistribution location and is not treated as the original dataset source. The dataset contains 7023 MRI image slices from four classes: glioma (1621), meningioma (1645), no tumor (2000), and pituitary tumor (1757). Only image files with supported extensions (.jpg, .jpeg, .png, .bmp) were included; non-image files and unsupported formats were excluded during manifest construction. The public dataset folders do not provide patient identifiers or patient-level grouping metadata. Therefore, patient-level statistics and patient-wise train/test separation could not be computed. The experiments use a stratified image-level split into 5617 training images, 703 validation images, and 703 held-out test images, with zero file overlap among the three subsets. The reported performance represents image-level benchmark performance under the stated public-dataset protocol, not patient-independent clinical validation (Tables 1, 2).

**Table 1:** Dataset class-count.

Class	Number of images
Glioma	1,621
Meningioma	1,645
No tumor	2,000
Pituitary	1,757
Total	7,023

**Table 2:** Original subset.

Original subset	Glioma	Meningioma	No tumor	Pituitary	Total
Training	1,321	1,339	1,595	1,457	5,712
Testing	300	306	405	300	1,311
Total	1,621	1,645	2,000	1,757	7,023

**Table 3:** Split table.

Split	Glioma	Meningioma	No tumor	Pituitary	Total
Training	1,297	1,315	1,600	1,405	5,617
Validation	162	165	200	176	703
Testing	162	165	200	176	703
Total	1,621	1,645	2,000	1,757	7,023

**Table 4:** Client-distribution table.

Client	Glioma	Meningioma	No tumor	Pituitary	Total
Client 0	258	254	326	286	1,124
Client 1	264	261	327	272	1,124
Client 2	249	259	319	296	1,123
Client 3	264	267	313	279	1,123
Client 4	262	274	315	272	1,123
Total	1,297	1,315	1,600	1,405	5,617

### Global backbone model

MobileNetV2 is used as the global feature extractor because of its computational efficiency and strong feature-representation capability for image classification tasks^35^. The backbone was initialized with ImageNet pretrained weights except for the first convolutional layer. Because the original MobileNetV2 input stem expects RGB input with kernel shape (32, 3, 3, 3), it was replaced by a single-channel convolution with shape (32, 1, 3, 3), preserving the same output channels, kernel size, stride, and padding. The replacement first convolution was randomly initialized using PyTorch’s default Conv2d initialization; no averaging or summation of the RGB pretrained kernels was applied. The final classification layer of MobileNetV2 was discarded, and the backbone output was transformed into a 1280-dimensional feature vector after global average pooling^35^.

During federated learning, only the MobileNetV2 backbone parameters are exchanged between the clients and the central server. The client-side MLP, SVM, and ELM meta-learners remain local and are not transmitted during federated aggregation^3,23^. Therefore, the meta-learners do not add communication payload beyond the shared backbone exchange (Tables 3, 4).

### Federated training procedure

Federated training proceeds through four repeated steps in each communication round:

(1) Global model distribution:

The server distributes the current global backbone model to each client.

(2) Local model training.

Each client trains the global backbone model on their own MRI images. They use a cross-entropy loss function with class weights to address class imbalance. To reduce local-update drift, each client also adds a proximal regularization term to keep the local model close to the global model. This reduces excessive divergence between local client updates and the global model.

(3) Client update submission:

Each client submits their updated global backbone model weights to the server.

(4) Model aggregation.

The server combines all the updates using a weighted average. The weighted average is then the new global backbone model for the next round.

This continues until the global backbone model has stable performance on all clients. After each communication round, the updated global backbone model is evaluated on a centralized validation set to compute accuracy, precision, recall, and F1-score. This ensures consistent and comparable assessment of convergence across rounds, avoiding variability introduced by client-specific validation distributions.

Mathematically, the global model update is defined as:

1 $$\:{\omega\:}_{global}=\:\varSigma_{k} \left(\frac{n_{k}}{N}\right) \cdot \omega_{k}$$

where $\:\boldsymbol{\omega\:}_{\boldsymbol{k}}$ represents the model parameters from client k, $\:\boldsymbol{n}_{\boldsymbol{k}}\:$is the number of samples at client k, and N is the total number of samples.

To reduce local-update drift, FedProx modifies the local objective function as:

2 $$\:{L}_{FedProx}\left({\omega\:}_{i}\right)=\:{L}_{local}\left({D}_{i},{\omega\:}_{i}\right)+\:\left(\frac{\mu\:}{2}\right)\:{\left|\left|{\omega\:}_{i}\:-\:{\omega\:}_{global}\right|\right|}^{2}$$

where µ = 0.0001 controls the strength of regularization, ensuring that local updates remain close to the global model and improving convergence stability.

(5) Local meta-learners.

During each federated round, after local backbone updates, each client uses the current global backbone to extract the embeddings of its features^35^. The embeddings are then passed to the local meta-learners:

- Multilayer Perceptron (MLP) for non-linear classification choices^9^.
- Support Vector Machine (SVM) for margin-based classification^9^.
- Extreme Learning Machine (ELM) for closed-form classification^17^.

The selection of MLP, SVM, and ELM as meta-learners is motivated by their complementary strengths in handling medical image feature embeddings. The MLP captures complex non-linear relationships in high-dimensional representations, enabling it to model intricate tumor patterns. The SVM provides robust margin-based classification, which is particularly effective in distinguishing classes with subtle variations commonly observed in brain tumor MRI data. The ELM offers fast training with strong generalization capability, making it suitable for efficient client-side deployment. These characteristics are particularly relevant in brain tumor MRI classification, where subtle inter-class variations (e.g., glioma vs. meningioma) require both nonlinear modeling (MLP), robust boundary separation (SVM), and efficient generalization under limited client data (ELM). The combination of these models enables better adaptation to local data variations and improves robustness.

The meta-learners enable each client to develop its own classifier without revealing its internal parameters^3^ because each meta-learner focuses on a different aspect of the local data, a combination of the meta-learners provides a more stable decision boundary than a single meta-learner^9^. The meta-learners are retrained at each federated learning round using feature embeddings extracted from the current global backbone model. As the backbone is progressively updated across rounds, the feature representations become increasingly discriminative. This enables the meta-learners to continuously adapt to the evolving feature space.

Compared to a purely sequential training strategy, where meta-learners are trained only after backbone convergence, the proposed co-adaptive approach allows meta-learners to continuously track improvements in feature representations, leading to better alignment with evolving feature spaces.

Although meta-learners are trained at each federated round to track the evolving feature space, the models used at inference correspond to the final round, where the global backbone has converged to its most informative representation. This ensures that meta-learners operate on a stable and high-quality embedding space at deployment, while still benefiting from progressive alignment during training.

The feature extraction process can be represented as:

3 $$\:{\mathrm{f}}_{{\uptheta\:}}:\mathrm{X}\to\:{\mathbb{R}}^{1280}$$

where the MobileNetV2 backbone maps input MRI images to a 1280-dimensional feature space.

For ensemble prediction, the final probability is computed as:

4 $$\:{\mathrm{p}}_{\mathrm{e}\mathrm{n}\mathrm{s}\mathrm{e}\mathrm{m}\mathrm{b}\mathrm{l}\mathrm{e}}=\frac{1}{3}\left({\mathrm{p}}_{\mathrm{M}\mathrm{L}\mathrm{P}}+{\mathrm{p}}_{\mathrm{S}\mathrm{V}\mathrm{M}}+{\mathrm{p}}_{\mathrm{E}\mathrm{L}\mathrm{M}}\right)$$

For reproducibility and easier comparison, the final meta-learner configurations are summarized in Table 5.

**Table 5:** Configuration of local meta-learners.

Component	Configuration	Hyperparameters	Justification
Input embedding	MobileNetV2 feature vector	1280 dimensions	Compact representation from global backbone
MLP	Linear(1280,128), ReLU, Dropout(0.3), Linear(128,4)	Adam, lr = 1e-3, batch size = 64, epochs = 20, cross-entropy	Nonlinear local classifier; dropout limits overfitting
SVM	RBF-kernel SVM with probability estimation	StandardScaler, C = 1.0, gamma=scale, probability=True	Margin-based nonlinear classifier with probability outputs
ELM	Closed-form ridge classifier on embeddings	lambda = 0.001	Fast deterministic classifier with regularized solve
Ensemble	Probability-level averaging	Equal weights	Avoids extra trainable fusion parameters

The hyperparameters were selected based on preliminary experimentation, convergence stability, and empirical validation to keep the local meta-learning stage lightweight across clients. Adam with lr = 1e-3 was used for the MLP because adaptive moment estimates are stable for high-dimensional embeddings and small neural classifiers. The 20-epoch setting allowed the lightweight MLP to converge without adding large local computation. The SVM used an RBF kernel with probability estimation for nonlinear decision modeling and probability-level averaging, while the ELM used ridge regularization with λ = 0.001 for numerical stability. Once selected, the same settings were fixed across all clients and experimental comparisons.

During meta-learner training, feature extraction is performed without gradient computation using torch.no_grad(), and the backbone operates in evaluation mode (model.eval()). Therefore, no gradient updates or fine-tuning of the backbone occur during this phase.

Feature embeddings are extracted from each client’s locally updated backbone model, and the meta-learners are trained on these pre-computed representations. The backbone is not involved in the optimization of meta-learners, ensuring a clear separation between feature extraction and local decision modeling. Specifically, this design ensures that: (i) the backbone remains unchanged during meta-learner training, (ii) feature extraction is consistent and reproducible across clients, and (iii) local personalization is achieved without additional communication or interference with global model updates.

(6) Ensemble-based global inference.

During evaluation, the global backbone is used (fully trained via federated learning) to extract feature embeddings from the input MRI samples. These embeddings are independently evaluated by the meta-learners at each client, where each client produces a probability distribution over the tumor classes. The final prediction is obtained by averaging these probability distributions across all clients^9^.

**Fig. 3:**
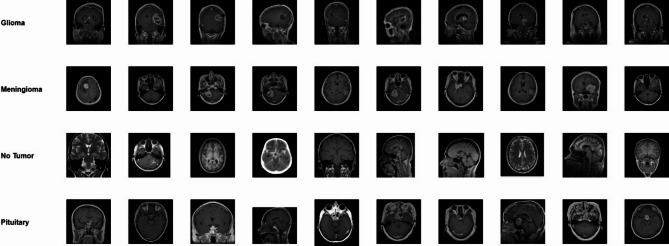
Four categories: glioma, meningioma, no tumor, and pituitary.

While this uniform element-wise averaging serves as a simple and efficient baseline consensus strategy, it implicitly assumes equal reliability across all clients. In practical multi-institutional settings, however, client datasets may differ in terms of data quality, sample size, and representativeness. As a result, equal weighting may underutilize more informative clients and amplify less reliable ones, potentially affecting overall diagnostic performance. Future work may explore adaptive aggregation strategies, such as confidence-weighted or performance-based averaging, to better handle such heterogeneity (Fig. 3).

This evaluation framework preserves privacy and improves performance:

- Privacy is maintained, as no raw data or model parameters are shared between clients.
- Performance benefits from aggregating diverse knowledge across multiple decentralized data sources, improving generalization^9^.

**Fig. 4:**
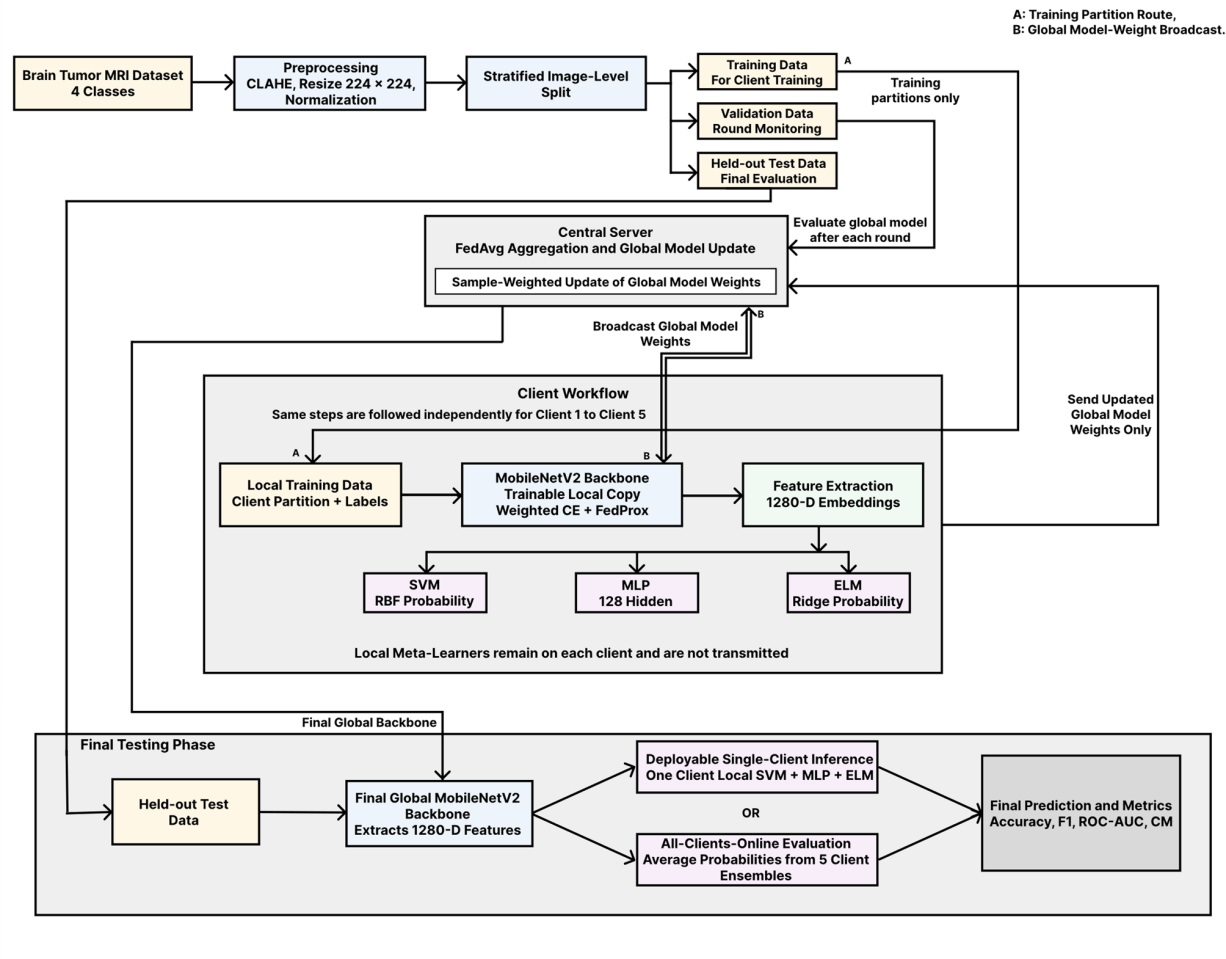
Flow of proposed methodology.

(7) Architectural summary.

The proposed architecture combines federated global representation learning with local decision adaptation. A shared MobileNetV2 backbone is optimized across five simulated clients using FedAvg aggregation with FedProx regularization, while each client trains local MLP, SVM, and ELM meta-learners on the 1280-dimensional embeddings extracted from its local training data. The resulting framework separates global feature learning from client-side classification, allowing the global model to benefit from collaborative training while keeping the meta-learners local and outside the federated communication process. During evaluation, the final global backbone extracts test embeddings, which are classified either by a single client’s local meta-learner ensemble or by the all-clients-online probability average.

(8) Training and inference protocol.

To ensure clarity and reproducibility, the complete training and inference pipeline is described as follows:

(1) Dataset Division:

The dataset is divided in a stratified manner to maintain class balance across three mutually exclusive subsets:

- Training Set: Approximately 80% of the dataset is used for model training and is distributed across five simulated clients using an approximately balanced round-robin assignment.
- Validation Set: Approximately 10% of the dataset is used as a centralized validation set to monitor global model performance after each federated round.
- Held-out Test Set: The remaining 10% of the dataset is reserved only for final evaluation and is never used during training, validation monitoring, hyperparameter selection, or meta-learner fitting.

(2) Federated backbone training:

The global MobileNetV2 backbone is initialized with ImageNet pretrained weights, except for the first convolutional layer, which is replaced with a randomly initialized single-channel convolution. Federated backbone optimization is conducted for 20 communication rounds. In each round, the server distributes the current global model to all clients, each client performs 2 local epochs using class-weighted cross-entropy with FedProx regularization (µ = 0.0001, learning rate = 1e-3, batch size = 16), and the returned model weights are aggregated using sample-weighted FedAvg. After each round, the updated global model is evaluated on the validation set using accuracy, precision, recall, and F1-score. Here, the 20 communication rounds and 2 local epochs refer to federated backbone optimization.

(3) Local meta-learner training:

After local backbone updates, each client extracts 1280-dimensional embeddings from its local data using the updated backbone in evaluation mode without gradient computation. The client then trains local MLP, SVM, and ELM meta-learners on these embeddings. The MLP is trained for 20 epochs using Adam with learning rate 1e-3; this 20-epoch setting applies only to the lightweight local meta-learner and is separate from the 20 federated communication rounds used for backbone optimization. The SVM uses an RBF kernel with probability estimation, and the ELM uses ridge regularization with λ = 0.001. For final deployment and testing, only the meta-learners trained in the final federated round are used.

(4) Inference procedure:

During inference, the final global MobileNetV2 backbone extracts a 1280-dimensional feature vector from each held-out test image. In the all-clients-online evaluation setting, this feature vector is evaluated by each client’s local MLP, SVM, and ELM meta-learners, and the resulting client-level probabilities are averaged according to the ensemble rule defined in Eq. (5). This setting evaluates cross-client ensemble diversity but requires all clients to participate at inference time. In the deployable single-client setting, a hospital uses the shared global MobileNetV2 backbone and only its own local MLP, SVM, and ELM meta-learners; therefore, no inter-client probability exchange, feature sharing, raw image sharing, or meta-learner parameter sharing is required during inference. This protocol maintains a clear separation between training, validation, and held-out testing and prevents file-level data leakage.

5 $$\:{p}_{final}\:=\:\left(\frac{1}{5}\right)\:\boldsymbol{\varSigma}_{k} \:\left(\frac{1}{3}\right)\left({p}_{{MLP}^{k}}+\:{p}_{{SVM}^{k}}+\:{p}_{{ELM}^{k}}\right)$$

To reduce overfitting and data leakage risk, the dataset was split into mutually exclusive training, validation, and held-out test subsets before model evaluation. The held-out test subset was not used during federated training, validation monitoring, hyperparameter selection, or meta-learner fitting. Data augmentation was applied only to training images, while validation and test images used deterministic preprocessing only. During meta-learner training, feature extraction was performed using model.eval() and torch.no_grad(), so the backbone was not updated by the meta-learner stage. Additional safeguards included dropout in the MLP, class-weighted cross-entropy, validation monitoring across communication rounds, repeated-run evaluation, confidence intervals, and paired statistical testing. Because the public dataset does not provide patient identifiers, the evaluation is an image-level benchmark and patient-level leakage cannot be fully ruled out; this limitation is explicitly acknowledged.

**Table 6:** Single-client inference.

Inference mode	Client	Accuracy (%)	Macro-F1 (%)	Weighted-F1 (%)	Requires inter-client probability exchange
All-clients-online probability average	All	99.15	99.16	99.15	Yes
Single-client local meta-ensemble	Client 0	98.86	98.89	98.86	No
Single-client local meta-ensemble	Client 1	98.72	98.72	98.72	No
Single-client local meta-ensemble	Client 2	99.00	99.01	99.00	No
Single-client local meta-ensemble	Client 3	97.87	97.82	97.87	No
Single-client local meta-ensemble	Client 4	98.15	98.12	98.15	No
Single-client mean ± SD	Clients 0–4	98.52 ± 0.49	98.51 ± 0.52	-	No

(9) Communication cost estimation.

Communication cost was estimated from the serialized model payload exchanged during federated training. In each communication round, the server sends the current global backbone to each client, and each client returns its locally updated backbone to the server. Therefore, for * K* clients, $\:R\:$communication rounds, and serialized backbone size * S* MB, the total cumulative communication cost is:

6 $$\:{C}_{\mathrm{t}\mathrm{o}\mathrm{t}\mathrm{a}\mathrm{l}}=2\times\:K\times\:R\times\:S$$

The per-round communication cost is:

7 $$\:{C}_{\mathrm{r}\mathrm{o}\mathrm{u}\mathrm{n}\mathrm{d}}=2\times\:K\times\:S$$

where the factor 2 accounts for both downlink and uplink communication. In this study,* K=5*, * R=20*, and the serialized MobileNetV2 backbone payload is * S=14.99*MB. Therefore:

$$\:{C}_{\mathrm{r}\mathrm{o}\mathrm{u}\mathrm{n}\mathrm{d}}=2\times\:5\times\:14.99=149.89\:\mathrm{M}\mathrm{B}$$ $$\:{C}_{\mathrm{t}\mathrm{o}\mathrm{t}\mathrm{a}\mathrm{l}}=2\times\:5\times\:20\times\:14.99=2997.83\:\mathrm{M}\mathrm{B}$$

The local MLP, SVM, and ELM meta-learners are trained locally and are not transmitted to the server; therefore, they do not add federated communication payload beyond the MobileNetV2 backbone exchange.

**Figure:**
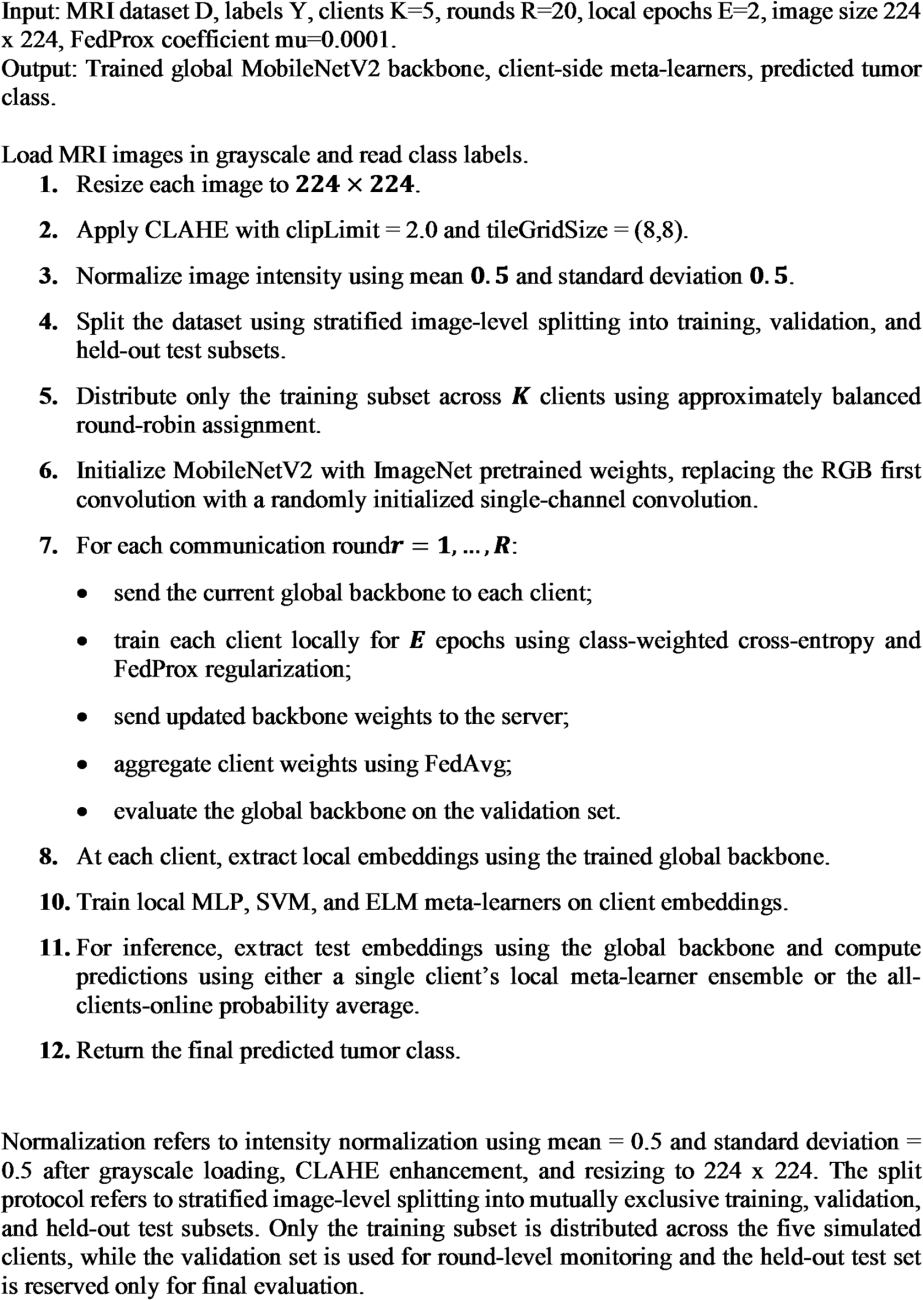
Algorithm.

## Results and discussion

### Global model performance across federated rounds

**Fig. 5:**
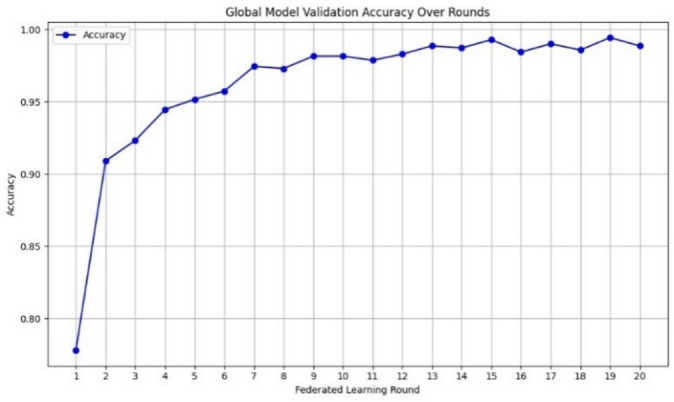
Global model validation accuracy.

**Fig. 6:**
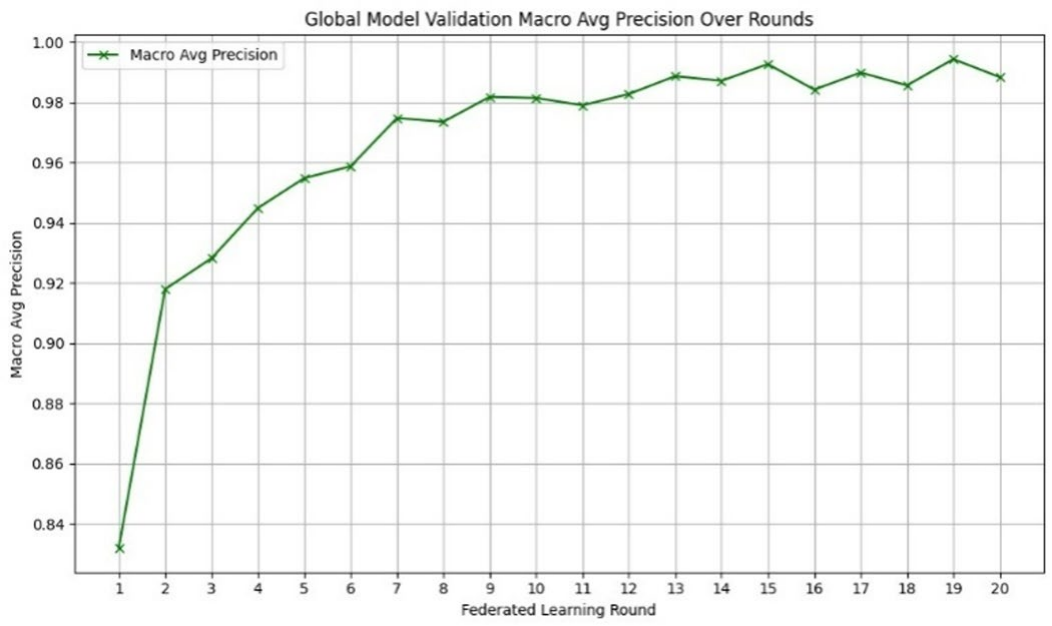
Global model validation precision.

**Fig. 7:**
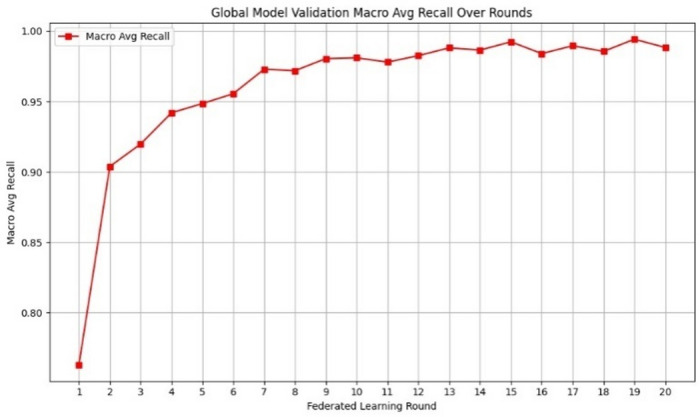
Global model recall.

**Fig. 8:**
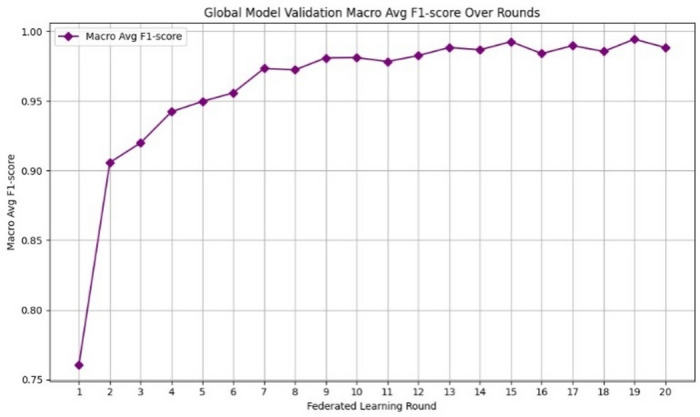
Global model F1-score.

**Fig. 9:**
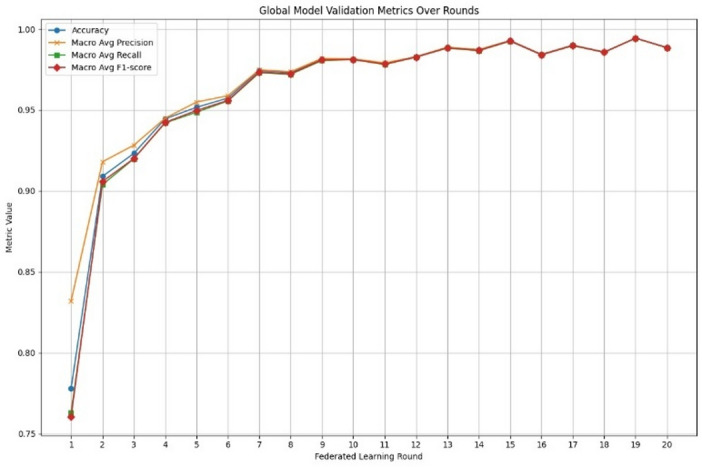
Global model validation metrics.

The performance of the global MobileNetV2 model was monitored after each round of communication to check how the training was progressing. Four key performance metrics were monitored: accuracy, precision, recall, and F1-score over 20 rounds, plotting each. All results presented in Figs. 5, 6, 7, 8 and 9 are obtained using the training and evaluation protocol described in Section IV-G. The global model is evaluated after each federated round on a centralized validation set to ensure consistent and comparable performance measurement.

1. Accuracy Progression (Fig. 5).

The accuracy increased rapidly in the initial rounds, indicating that the global model was learning quickly from the distributed MRI images, even with non-identically distributed data. After Round 8, it stabilized, indicating that the critical features were now locked in. The slight increases thereafter are merely fluctuations due to changes in the client data.

2. Precision progression (Fig. 6).

Precision increased steadily, indicating that the model was improving its ability to correctly classify tumor types while minimizing false positives. Once it stabilized, precision remained high, indicating that the model was robust across clients.

3. Recall Progression (Fig. 7).

Recall also increased steadily. The positive trend indicates that the federated model was successfully extracting class-specific features despite the non-identically distributed data among clients.

4. F1-Score Progression (Fig. 8).

Because F1-score is a measure that balances precision and recall, the positive trend here further indicates that the federated learning process was successful. The high scores were achieved early on and remained relatively stable thereafter, indicating that the global model was robust during federated learning.

Each of the four graphs (Figs. 5, 6, 7 and 8) provides a comprehensive understanding of how the global model was progressing. The positive trend and later stabilization of the four metrics indicate a successful convergence of the federated learning process.

It is important to note that the reported performance metrics correspond to the global model evaluated on a centralized validation set rather than individual client models. This approach ensures a fair and stable assessment of convergence behavior across federated rounds. While client-wise performance analysis can provide additional insights in highly non-IID scenarios, the balanced data partitioning adopted in this study ensures that global evaluation remains representative.

### Combined global validation metrics

To illustrate the relationship between accuracy, precision, recall, and F1-score, a combined graph was created (Fig. 9). This graph illustrates how all these values progress together during training. They all progress together, with none of them falling behind, indicating that the model did not overfit one class of tumors more than the others and performed equally well on all classes.

The combined graph also shows the effect of FedAvg aggregation and proximal regularization. The values level off after the midpoint, indicating that the additional stability regularization had the effect of keeping the global updates stable (Table 7).

**Table 7:** Global model validation metrics across rounds.

Rounds	Global model accuracy	Global precision	Global recall	Global F1 score
1	0.7781	0.8331	0.7781	0.7705
2	0.9090	0.9191	0.9090	0.9091
3	0.9232	0.9297	0.9232	0.9225
4	0.9445	0.9459	0.9445	0.9441
5	0.9516	0.9555	0.9516	0.9516
6	0.9573	0.9598	0.9573	0.9573
7	0.9744	0.9754	0.9744	0.9744
8	0.9730	0.9741	0.9730	0.9731
9	0.9815	0.9821	0.9815	0.9816
10	0.9815	0.9819	0.9815	0.9816
11	0.9787	0.9792	0.9787	0.9787
12	0.9829	0.9833	0.9829	0.9830
13	0.9886	0.9887	0.9886	0.9886
14	0.9872	0.9875	0.9872	0.9872
15	0.9929	0.9929	0.9929	0.9929
16	0.9844	0.9847	0.9844	0.9844
17	0.9900	0.9901	0.9900	0.9901
18	0.9858	0.9860	0.9858	0.9858
19	0.9943	0.9943	0.9943	0.9943
20	0.9886	0.9887	0.9886	0.9886

### Final test accuracy and global evaluation metrics

After 20 federated rounds of training, the final global backbone model was evaluated on the held-out test set. Using the client-side meta-learner ensemble for prediction, the system achieved a maximum observed accuracy of 99.57%. The final evaluation also showed strong precision, recall, and F1-score, indicating that the performance was not limited to accuracy alone and that the model maintained balanced classification behavior across tumor classes.

This is important because F1-score reflects the balance between precision and recall in the four-class tumor classification task, while ROC-AUC confirms the model’s class-discrimination capability beyond accuracy (Table 8).

**Table 8:** Global model evaluation metrics across rounds.

Tumor class	Precision	Recall	F1-Score	Support
Glioma	0.9938	0.9938	0.9938	162
Meningioma	0.9940	1.0000	0.9970	165
No Tumor	1.0000	0.9900	0.9950	200
Pituitary	0.9943	1.0000	0.9972	176
Accuracy			0.9957	703
Macro Avg	0.9955	0.9960	0.9957	703
Weighted Avg	0.9957	0.9957	0.9957	703

### Confusion matrix interpretation (Fig. 10)

The confusion matrix is a way of analyzing how well the model performed on each class. Most of the predictions are on the diagonal, which means that the predictions are mostly correct. There are not many predictions that are incorrect, and these are mostly for types of tumors that look similar.

The confusion matrix reveals:

- High sensitivity for each type of tumor.
- Low false positives and false negatives.
- A balanced pattern and no clear dominance of any class.

All of this is evidence that the learning of the model was not affected by the class imbalance and the differences between the clients.

**Fig. 10:**
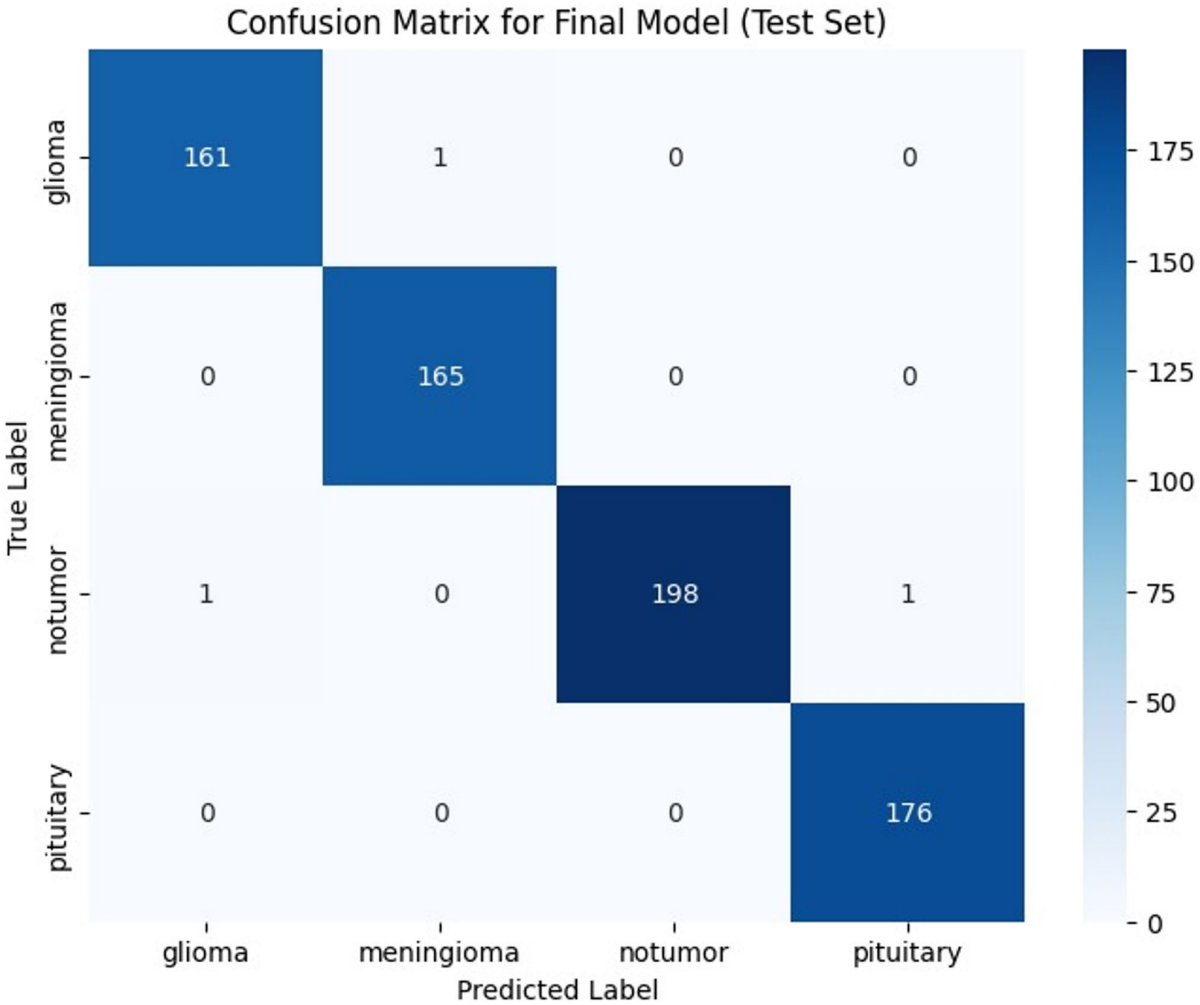
Confusion matrix for final model.

### Discussion and insights

Here are a few points that can be taken away from the results:

- Early convergence indicates that MobileNetV2 is a good option for feature extraction in this federated MRI environment.
- The values remain constant, indicating that the backbone network maintained a consistent level of performance despite the variations in the clients.
- The high level of final test accuracy, together with strong precision, recall, F1-score, and ROC-AUC values, indicates that the combination of federated representation learning and meta-learning is beneficial.
- The confusion matrix reveals that there is a distinct separation between the classes and that there is no overlap between the classes of tumors.
- The ensemble inference result reveals that the knowledge from multiple clients is beneficial in avoiding overfitting to a single data silo.

The combination of federated learning and meta-learning provides strong diagnostic accuracy while preserving privacy in the evaluated image-level benchmark. Although the experiments are conducted in a controlled setting with balanced sample allocation across clients, the consistent macro-average precision, recall, and F1-score indicate balanced performance across tumor classes.

In the current design, meta-learners are retrained at each federated round using the updated backbone features, enabling co-adaptation between representation learning and local decision boundaries. While this introduces a non-stationary feature space during training, only the final-round meta-learners are used at inference, ensuring stability. For environments with significant distribution shift over time, future work may explore periodic re-training of meta-learners using a frozen backbone to balance stability and adaptability without incurring additional federated communication cost.

The co-adaptive training of meta-learners at each federated round allows them to align with progressively improving feature representations. While this introduces a non-stationary feature space during training, the use of final-round meta-learners ensures stable and consistent inference.

Adaptive aggregation strategies, such as confidence-weighted averaging, performance-based client weighting, or Bayesian model combination, represent promising extensions to improve robustness under heterogeneous client conditions and are identified as directions for future work.

### Error analysis and failure cases

To better understand the limitations of the proposed framework, misclassifications are analyzed based on the confusion matrix obtained from the test set evaluation.

The following failure patterns are observed:

- *Meningioma–Glioma Confusion* The most frequent misclassification occurs between meningioma and glioma cases. These tumor types often exhibit overlapping characteristics such as irregular boundaries, surrounding edema, and similar intensity distributions in MRI images. As a result, the feature representations extracted by the backbone may not fully capture subtle distinctions between these classes.
- *Small or Micro-Lesion Misses*: Cases where the tumor occupies a very small region of the image are occasionally misclassified. Although CLAHE enhances contrast, very small lesions may not be sufficiently emphasized after resizing to 224 × 224 resolution, leading to missed detections.
- *No-Tumor False Positives* A small number of healthy samples are incorrectly classified as tumors. This typically occurs when normal anatomical structures, such as ventricles or sulci, exhibit intensity patterns similar to tumor-like features in the learned feature space.

These observations highlight the limitations of the current framework, particularly in handling subtle, small-scale, or ambiguous tumor characteristics. Future work will focus on incorporating higher-resolution inputs and attention-based mechanisms to improve sensitivity to such challenging cases. Additionally, qualitative visualization of misclassified MRI samples will be explored for deeper clinical interpretability.

### Robustness across repeated runs

To assess sensitivity to data splitting, client assignment, model initialization, and mini-batch ordering, the proposed framework and the strongest internally controlled federated baseline were evaluated across four repeated runs. The mean, standard deviation, and 95% confidence interval are summarized in Table 9, and paired statistical tests are reported in Table 10.

The 95% confidence interval was reported to quantify the uncertainty around the repeated-run mean accuracy and to avoid relying only on a single point estimate. This interval provides a standard estimate of the plausible range of mean performance under repeated experimental runs with different random seeds.

**Table 9:** Multi-seed summary results.

Model	Accuracy mean ± SD (%)	95% CI for accuracy (%)	Macro-F1 mean ± SD (%)	Weighted-F1 mean ± SD (%)
CNN+FedAvg+FedProx	86.13 ± 2.35	82.39–89.88	85.55 ± 2.92	86.02 ± 2.75
Proposed MobileNetV2 + FedAvg+FedProx+MetaLearners	99.29 ± 0.20	98.97–99.61	99.28 ± 0.18	99.29 ± 0.20

The paired t-test shows a statistically significant improvement across the four matched seeds. The Wilcoxon signed-rank test is not significant because *n* = 4 is very small.

**Table 10:** Paired statistical tests.

Metric	Mean difference	Paired t-test *p*	Wilcoxon *p*
Accuracy	13.16% points	0.00184	0.125
Macro-F1	13.73% points	0.00292	0.125
Weighted-F1	13.27% points	0.00274	0.125

## Comparative analysis

In this section, the proposed method is compared with existing approaches. In addition to federated learning-based approaches, centralized deep learning models are considered to highlight the effectiveness of the proposed framework in terms of accuracy, F1-score, ROC-AUC, privacy preservation, and communication cost.

To demonstrate how effective the proposed system is, the proposed system is compared with a number of deep learning models and federated learning models that are currently used by people for the classification of brain tumors. The literature-context accuracy comparison is given in Table 11, while the corresponding accuracy comparison plot is shown in Fig. 12.

Traditional CNN models such as VGG16, ResNet, and other similar models perform well when they have access to centralized data because they are able to extract a number of features from the data. However, they require access to big centralized datasets. Since these models are centralized, they are not very effective when the data is distributed across different institutions.

Recent approaches based on texture features, multiple data streams, or transformers have demonstrated strong accuracy in centralized settings. However, many of these approaches do not directly address privacy-preserving distributed training or communication cost.

The FedAvg algorithm is applied in federated learning for a medical classification problem. The algorithm allows the model to train without viewing the entire data at once, but it performs poorly when the data from the clients is of different sizes or distributions. Without personalization or stabilization, the global model will represent the data of the largest clients or not generalize well to the rare cancers.

Unlike conventional global-model FL, the proposed framework integrates shared feature learning with local decision adaptation. The MobileNetV2 backbone learns global MRI representations, while the client-side MLP, SVM, and ELM meta-learners model local decision boundaries without transmitting meta-learner parameters. The deployable single-client inference results in Table 6 show that individual clients retained strong local performance using only their own meta-learner ensemble, supporting the practical hospital-side inference setting.

The ablation study in Table 16; Fig. 14 further clarifies the source of the performance gain. MobileNetV2 is the main contributor to representation quality, while the local meta-learner ensemble provides additional decision-level refinement. Therefore, the proposed contribution is not that all components contribute equally, but that the decoupled backbone-plus-local-meta-learner design combines strong shared representation learning with lightweight local personalization.

**Table 11:** Literature-context accuracy comparison.

Ref. No	Authors	Model	Accuracy	Dataset	Year
^23^	N. Sivakumar et al.	CNN+FedAvg+FedProx	97.19%	Brain Tumor MRI Dataset, Kaggle; 7,023 images; 4 classes	2025
^38^	Ullah et al.	Gabor Filter + ResNet50 + SVM	95.73%	Brain Tumor MRI Dataset, Kaggle; 7,023 images; 4 classes	2023
^25^	M. Ravinder et al.	GCNN	95.01%	Brain Tumor Classification (MRI), Kaggle, 3264 images; 4 classes	2023
^24^	S. Das et al.	CNN	94.39%	Brain Tumor Dataset, Figshare Cheng, 3064 images; 3 classes	2019
^26^	Liu D et al.	G-ResNet	95%	Brain Tumor Dataset, Figshare Cheng, 3064 images; 3 classes	2019
^39^	Ge C et al.	Multi-stream 2D-CNN model	88.82%	TCGA-LGG and TCGA-GBM, BraTS; 4 Modalities	2020
^12^	Cheng Y et al.	ConvCaps	93.50%	Brain Tumor Dataset, Figshare Cheng, 3064 images; 3 classes	2019
^13^	Afshar P et al.	BayesCap	73.90%	Brain Tumor Dataset, Figshare Cheng, 3064 images; 3 classes	2020
^40^	Abiwiananda N et al.	Custom CNN	84.19%	Brain Tumor Dataset, Figshare Cheng, 3064 images; 3 classes	2019
41	Naser M.A et al.	VGG16	89%	TCGA-LGG, Cancer Imaging Archieve, 3929 images; 2 classes	2020
42	S. Bhadauirya et al.	CNN + FL	96%	Not reported in accessible metadata	2023
^43^	S. Hossain et al.	IVX16	96.94%	Brain Tumor Classification (MRI), Kaggle, 3264; 4 Classes	2024
-	**Proposed Model**	**MobileNetV2 + Meta Learners+FedAvg+FedProx**	**99.57%**	**Brain Tumor MRI Dataset**,** Kaggle; 7**,**023 images; 4 classes**	**2026**

**Table 12:** Internally controlled baseline comparison under the same dataset, preprocessing, image-level split, and federated protocol.

Model	Setting	Accuracy (%)	Macro-F1 (%)	Weighted-F1 (%)
Centralized MobileNetV2	Pooled centralized training	97.87	97.81	97.87
CNN+FedAvg	Federated, 5 clients, 20 rounds	87.91	87.66	87.95
CNN+FedAvg+FedProx	Federated, 5 clients, 20 rounds	88.76	88.67	88.97
Proposed MobileNetV2 + FedAvg+FedProx+MetaLearners	Federated, 5 clients, 20 rounds + local meta-learners	99.15	99.16	99.15

To complement accuracy and F1-score comparison, ROC-AUC analysis was performed for the proposed framework and the internally controlled baselines using the same dataset, preprocessing, image-level split, and evaluation protocol. ROC curves were computed using a one-vs-rest strategy for the four-class classification task. Because sample-level probability outputs are not available for the literature-only baselines in Table 11, ROC-AUC comparison is restricted to the internally reimplemented models (Tables 12, 13, 14, 15).

**Fig. 11:**
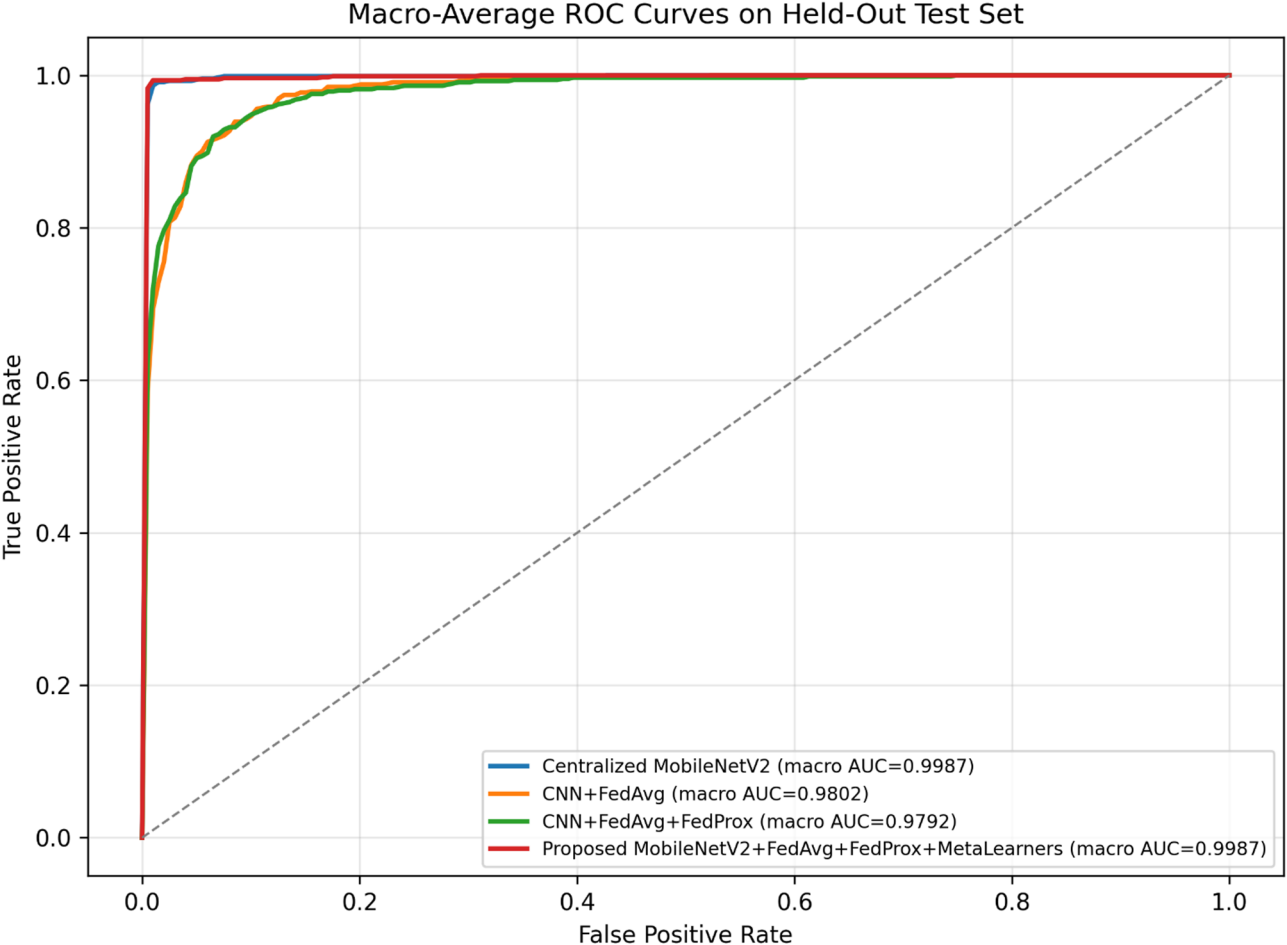
ROC curves for the proposed model and internally controlled baselines.

**Table 13:** Macro and weighted ROC-AUC comparison for internally controlled models.

Model	Macro AUC	Weighted AUC
Centralized MobileNetV2	0.998731	0.998698
CNN+FedAvg	0.980164	0.981138
CNN+FedAvg+FedProx	0.979161	0.980196
Proposed MobileNetV2 + FedAvg+FedProx+MetaLearners	0.998690	0.998598

**Table 14:** Class-wise ROC-AUC for the proposed model.

Class	ROC-AUC
Glioma	0.999030
Meningioma	0.999561
No tumor	0.996581
Pituitary	0.999590

The proposed framework achieved a macro-average ROC-AUC of 0.998690 and a weighted-average ROC-AUC of 0.998598, which is close to the centralized MobileNetV2 baseline and higher than the CNN-based federated baselines. This indicates that the proposed method preserves strong class-discrimination capability under the federated protocol while avoiding centralized training (Fig. 11).

**Fig. 12:**
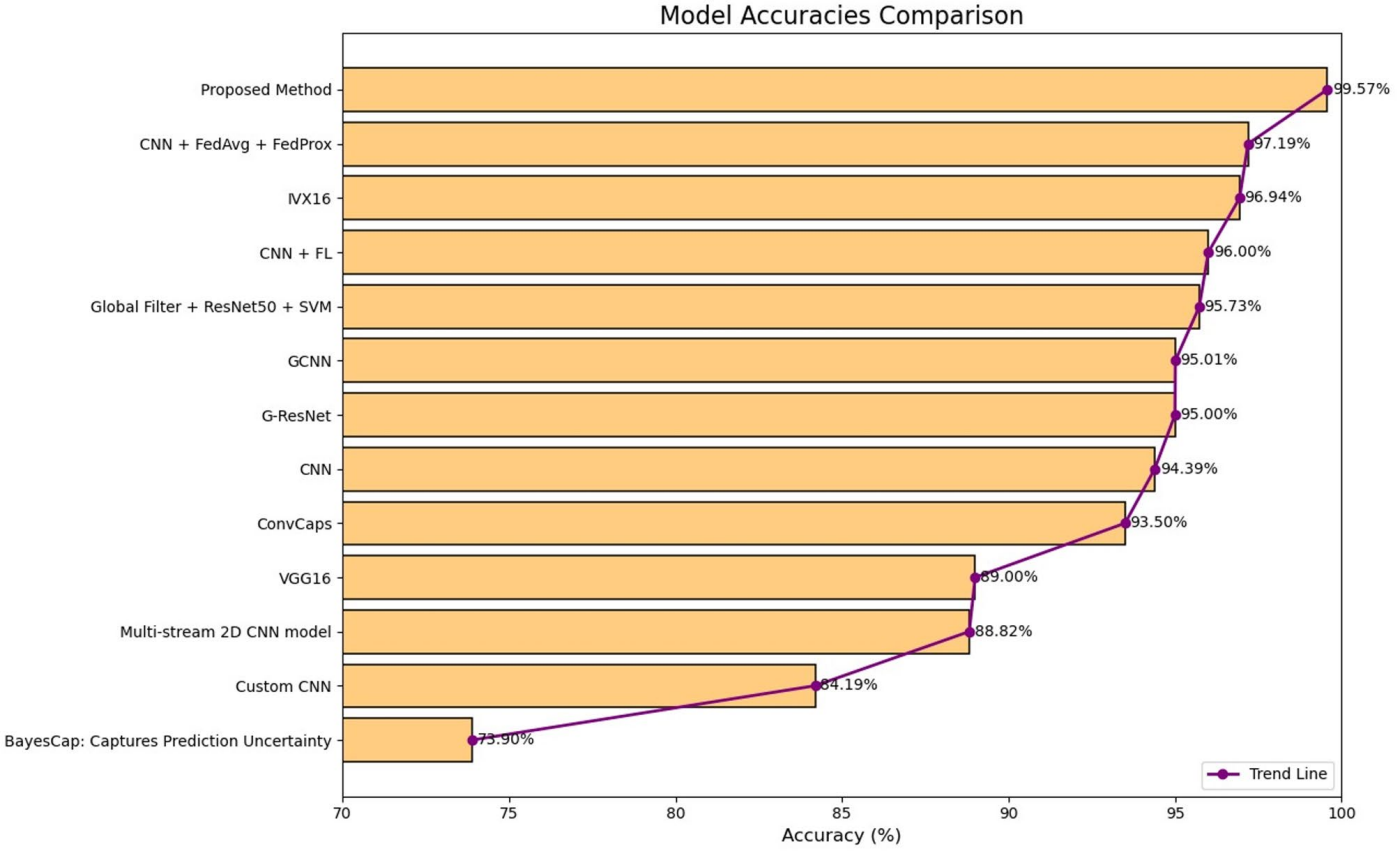
Accuracy comparison of proposed model.

**Table 15:** Communication cost.

Model	Parameters	Serialized payload per client per direction (MB)	Downlink per round, 5 clients (MB)	Uplink per round, 5 clients (MB)	Total per round (MB)	Total over 20 rounds (MB)	Reduction vs. ResNet-50 (%)
MobileNetV2 backbone + local meta-learners	3,868,100	14.99	74.95	74.95	149.89	2,997.83	83.34
ResNet-50 backbone baseline	23,509,956	89.98	449.91	449.91	899.82	17,996.32	-

**Fig. 13:**
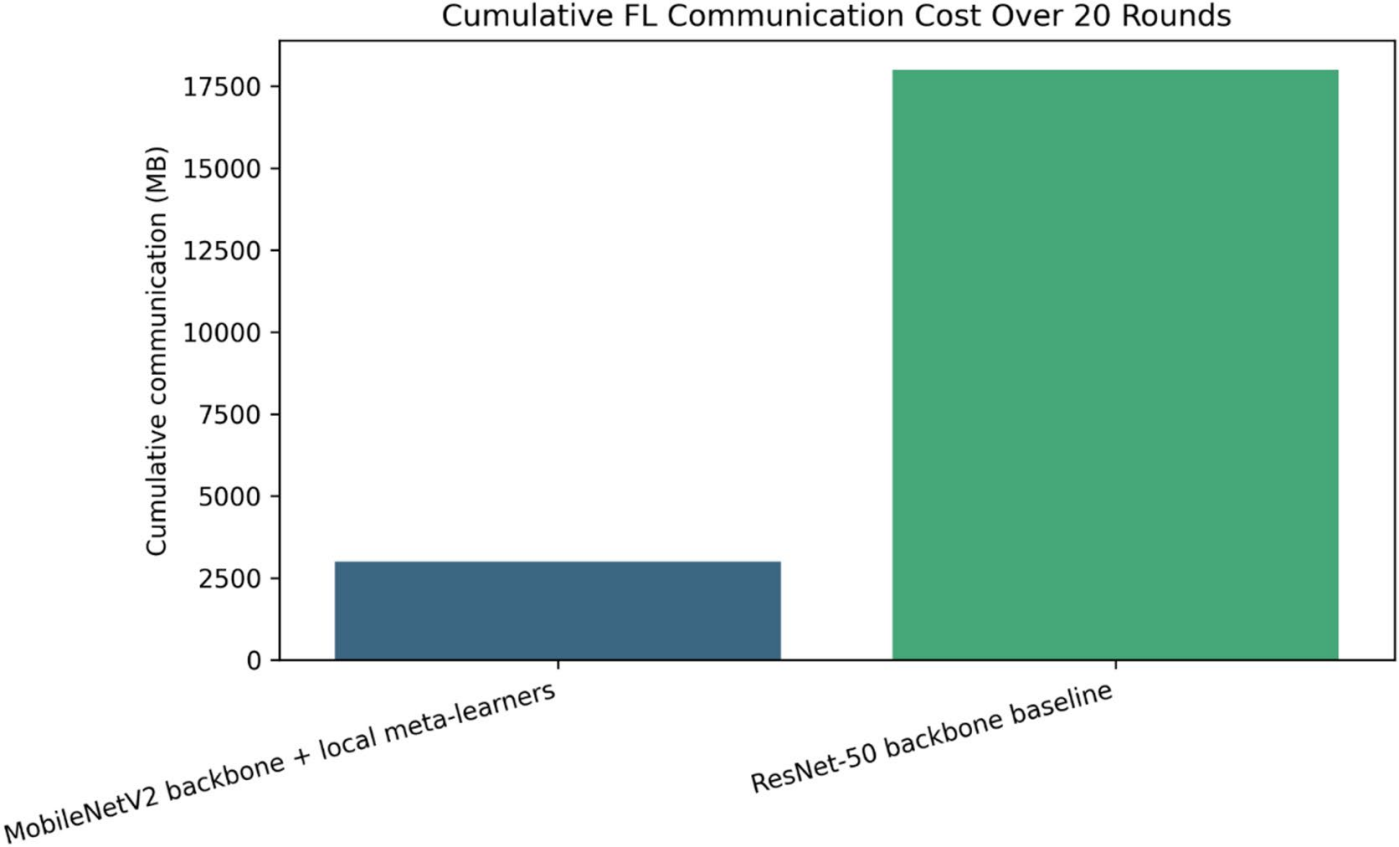
Communication cost comparison.

### Ablation study

An ablation study was conducted to isolate the contribution of the backbone architecture, FedProx regularization, and local meta-learner components. The CNN+FedAvg+FedProx baseline achieved 88.76% accuracy, whereas replacing the CNN with MobileNetV2 increased the backbone-only accuracy to 98.58%, showing that the lightweight pretrained backbone is the dominant contributor to representation quality. Under the present class-balanced near-IID client split, removing FedProx did not reduce accuracy; therefore, FedProx is interpreted as a local-update stability regularizer rather than the main source of performance gain in this experimental setting. For the meta-learners, the results show that the components do not contribute equally: MLP was the strongest individual classifier, SVM provided a competitive nonlinear margin-based classifier, and ELM showed limited standalone performance but provided complementary value in the MLP + ELM and full ensemble settings. The full MLP + SVM+ELM ensemble achieved 99.15% accuracy and 99.16% macro-F1 (Fig. 13).

**Table 16:** Ablation study of backbone, FedProx, and local meta-learner contributions.

Model/Variant	Ablation Focus	Accuracy (%)	Macro-F1 (%)	Weighted-F1 (%)	Interpretation
CNN+FedAvg+FedProx	Backbone baseline	88.76	88.67	88.97	CNN-based FL baseline
MobileNetV2 + FedAvg+FedProx backbone-only	Backbone contribution	98.58	98.58	98.58	MobileNetV2 substantially improves representation quality
MobileNetV2 + FedAvg backbone-only	FedProx contribution	99.00	99.02	99.00	FedProx mainly supports local-update stability under this near-IID split
MobileNetV2 + FedAvg+MetaLearners	FedProx removed	99.00	99.02	99.00	Meta-learner stage remains strong without FedProx
MobileNetV2 + FedAvg+FedProx + MLP	Meta-learner component	99.00	99.03	99.00	Strongest individual local meta-learner
MobileNetV2 + FedAvg+FedProx + SVM	Meta-learner component	98.44	98.45	98.44	Competitive nonlinear margin-based classifier
MobileNetV2 + FedAvg+FedProx + ELM	Meta-learner component	50.92	46.65	46.43	Limited standalone performance in this setting
MobileNetV2 + FedAvg+FedProx + MLP+SVM	Meta-learner pair	99.00	99.03	99.00	Similar to MLP alone
MobileNetV2 + FedAvg+FedProx + MLP+ELM	Meta-learner pair	99.15	99.16	99.15	Best paired combination
MobileNetV2 + FedAvg+FedProx + SVM+ELM	Meta-learner pair	98.44	98.46	98.43	Limited by weaker ELM/SVM pairing
MobileNetV2 + FedAvg+FedProx + MLP+SVM + ELM	Full proposed ensemble	99.15	99.16	99.15	Final local meta-learner ensemble

**Fig. 14:**
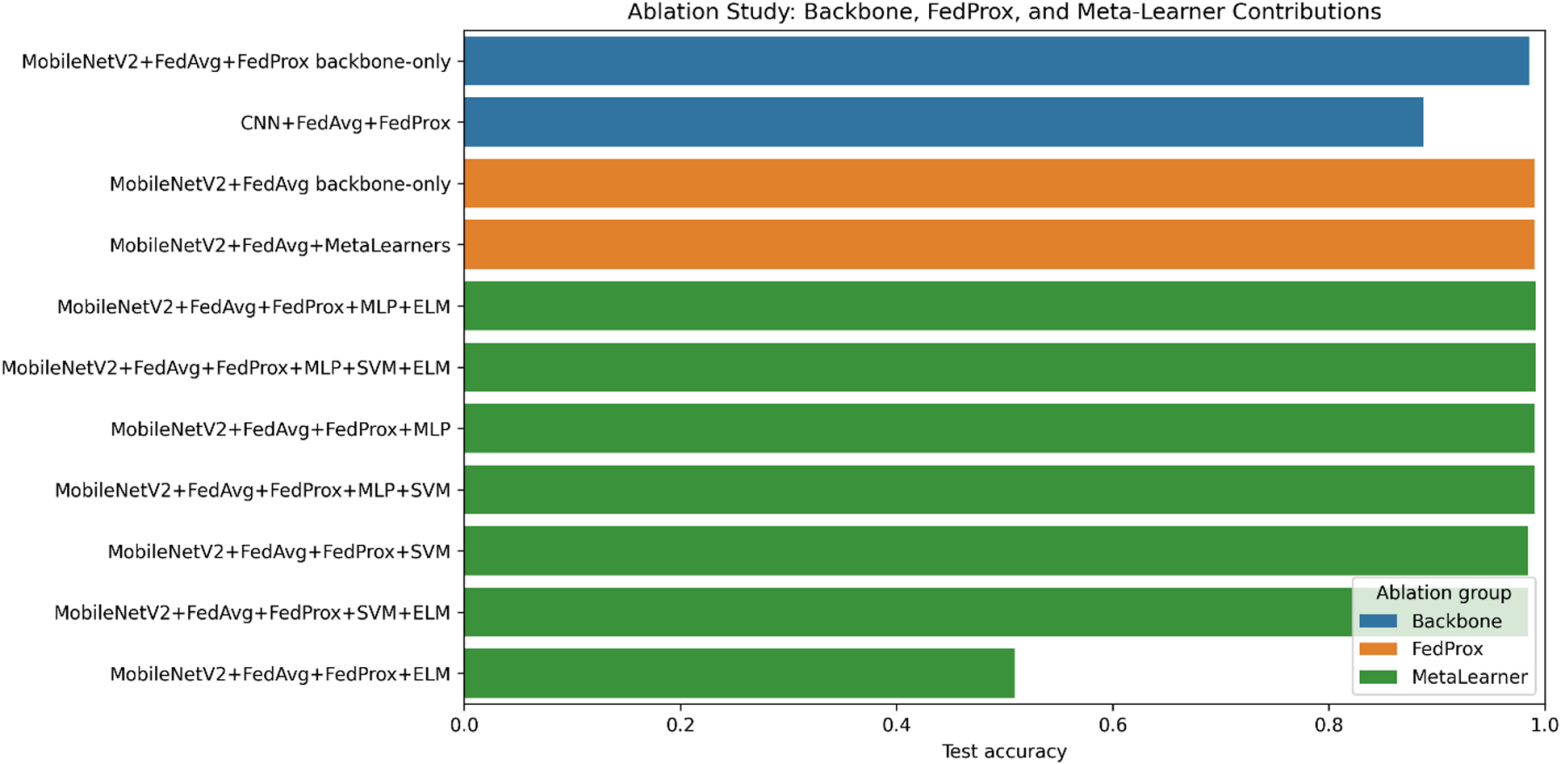
Ablation study showing test accuracy contributions of the backbone, FedProx, and local meta-learner components.

## Limitations and future work

### Limitations

This study is limited by the use of a public image-slice dataset without patient identifiers. Consequently, the train/validation/test split is image-level rather than patient-level, and subject-level leakage cannot be fully ruled out. The simulated client assignment is class-balanced and near-IID at the label level, so the present results should not be interpreted as a severe non-IID hospital stress test. Under this near-IID setting, the ablation study shows that FedProx does not improve accuracy over FedAvg, although it remains a stabilizing proximal regularizer. Future work should evaluate patient level multi-site datasets, explicit Dirichlet label-skew partitions, scanner/site heterogeneity, and real cross-silo deployment constraints.

### Future work

Future work will evaluate the framework with real-world hospital partners to determine how it can be applied in different ways at each hospital. We can explore the impact of explicit non-IID conditions, such as label distribution and data size, to better simulate real-world applications. Incorporating techniques for interpretability may increase adoption and ease of use in clinical settings. Using communication-efficient federated learning algorithms, such as sparse or quantized updates, may reduce the training time. Weighted or adaptive ensemble strategies may help improve the combination of local predictions when data quality and size are different for each client. Extending the application of the model to 3D MRI or other types of imaging is also a good direction.

Future work will focus on advancing the proposed framework through several key directions. First, dynamic or continual learning strategies will be explored to enable co-adaptation between the backbone and meta-learners, thereby improving real-time responsiveness to evolving local data distributions. Additionally, a comprehensive client-wise performance analysis under highly heterogeneous data settings will be conducted to further assess and enhance personalization capabilities. To gain deeper qualitative insights into model limitations, visualization of misclassified MRI samples will also be undertaken. Finally, the study will investigate the trade-offs between privacy and robustness, with particular emphasis on techniques such as weight-space noise injection to strengthen privacy preservation during federated training, as suggested by Darzi et al.^44^.

## Conclusion

This paper presents a decoupled federated-personalized learning framework for brain tumor classification. It adopts a globally shared MobileNetV2 backbone and locally trained MLP, SVM, and ELM meta-learners. This enables collaborative model development across simulated medical clients without sharing original MRI images, supporting privacy-preserving training. FedProx was used as a proximal regularization component to support local-update stability during federated optimization, while the local meta-learners remained client-side and were not transmitted during aggregation.

During global optimization, the shared MobileNetV2 representation was progressively updated across communication rounds, and the local meta-learners adapted to the extracted embedding space. The proposed framework achieved a maximum observed accuracy of 99.57% and a repeated-run accuracy of 99.29% +/- 0.20% on the Brain Tumor MRI Dataset, with consistently strong F1-score performance across repeated evaluation. Internally controlled baselines confirmed that the MobileNetV2 backbone and local meta-learner stage substantially improved accuracy and macro-F1 over CNN-based FL baselines under the same preprocessing and split protocol. ROC-AUC analysis further showed near-centralized discriminative performance, while communication-cost analysis showed an 83.34% payload reduction relative to a ResNet-50 backbone under the same federated protocol.

These results show that the combination of distributed feature learning and local meta-learning is an efficient and communication-conscious approach for image-level medical classification benchmarks. However, because the dataset does not provide patient identifiers, the present evaluation is based on image-level splitting rather than patient-level validation. In addition, the simulated client partition is class-balanced and near-IID at the label level. Future work should evaluate the framework using patient-level multi-institutional datasets, explicit Dirichlet label-skew partitions, scanner/site heterogeneity, and real cross-silo medical deployment settings.

## Data Availability

The datasets generated and/or analysed during the current study are available in the GitHub repository, and can be accessed through the given link- [https://github.com/Ankitesh999/brain\_tumor\_dataset.git](https:/github.com/Ankitesh999/brain_tumor_dataset.git).
